# Analysis of safety risks in mixed driving of manual and automawtic vehicles: multiple perspectives

**DOI:** 10.1371/journal.pone.0320834

**Published:** 2025-05-15

**Authors:** Yaqin He, Jun Xia, Jiayin Dai

**Affiliations:** School of Automobile and Traffic Engineering, Wuhan University of Science and Technology, Wuhan, Hubei, China; Nantong University, CHINA

## Abstract

To improve traffic safety in mixed traffic involving human-driven and autonomous vehicles, this study explored safety risk factors from multiple perspectives. Based on crash reports involving autonomous vehicles (AVs) in the California, United States, the XGBoost algorithm and Shapley additive explanations (SHAP) analysis were used to investigate the factors affecting accident severity. Association rule mining was employed to analyze the factors contributing to emergency braking events, based on field data from driverless taxi operations in China. Additionally, using data collected from questionnaires, the risk perception factors of different traffic participants were examined using the average degree of aggressiveness method. The results of three aspects analysis revealed that risk factors associated with mixed traffic were concentrated in areas such as weekdays, road sections, multiple lanes, roads with central medians, lack of control, and adverse environments. Finally, some safety improvement suggestions are recommended.

## 1 Introduction

An emerging technology, autonomous vehicles (AVs), has the potential to revolutionize the transportation industry by executing driving tasks, enhancing traffic efficiency, and improving traffic safety [1]. According to a report of the National Highway Traffic Safety Administration (NHTSA), 94% of serious car accidents in the U.S. involve human-driver-related factors. These factors include dangerous driving, distraction, speeding, and illegal driving [2]. Hence, the introduction of AV technology is anticipated to prevent traffic accidents and fatalities significantly by reducing human error, especially with fully automatic vehicles which do not need human driver intervention in any situation. [3] However, mixing AVs into a traffic system may pose new traffic safety risks associated with poorly maintained road markings, light reflections affecting the vehicle sensors, AV communication faults, cybersecurity, disengagements, etc. [4]. In addition, AV technology development trends suggest that AVs will inevitably share roads with other road users, such as conventional vehicle drivers, and pedestrians and cyclists, for a long time [5]. Although AVs largely benefit drivers, as their roles are replaced by those of automation system, the profits of AVs for other road users are not yet clear, and the challenges regarding the interaction between AVs and other road users are already foreseeable [6]. Notably, both the annual number of miles traveled by autonomous vehicles (AVMTs) and the number of AV accidents on public roads in California have increased every year between 2015 and 2022, except for a possible decline in 2020 due to the COVID-19 pandemic [7]. Moreover, crashes involving AVs are caused primarily by complicated interactions between AVs and conventional vehicles [8]. A previous report has shown that pedestrians may take advantage of AVs to the point that they can bully them, thus resulting in traffic crashes [9]. Therefore, clarifying the influencing factors contributing to AV accidents and recognizing potential risk factors in mixed traffic operations are crucial to improve the safety of AVs and other traffic participants.

Research on the safety risk of AVs has focused on various contributing factors affecting the collision types and severity of AV crashes on the basis of limited AV-related crash reports, but has overlooked the subjective risk perceptions of other road users when they interact with AVs, which could play a vital role in their interaction [10]. It is critical to understand the risk perceptions and driving behaviors of road users facing AVs to guide the safe and effective integration of AVs on roads [11]. Additionally, crash reports involving AVs generally contain basic information about the crash, such as the collision type, severity, accident location, weather, etc., without a detailed description of the accident cause and roadway features, which are very important for analyzing the AV accident mechanism and improving the safety of AVs [1]. To address this gap, this study investigated the safety risk factors in mixed traffic operations from multiple perspectives, including real AV accident data, abnormal driving behaviors and road users’ subjective perceptions. This study provides engineers, designers and managers with insight into the safety aspects of automated driving. The main contributions are as follows.

1) The factors contributing to the severity of AV accidents were quantitatively analyzed via the XGBoost model. In addition to the factors included in the AV crash reports in California, we used Google Street View to extract more detailed roadway features data as supplemental variables, making the analysis more comprehensive.
2) To analyze the risk of AV accidents in different countries and understand the possible causes of accidents and because there are currently no open data on AV accidents in China, we investigated the abnormal driving behaviors of AVs in Wuhan, China, and explored the factors leading to abnormal behaviors to reduce the likelihood of occurrence.
3) In addition to the risk analysis of objective data, this study innovatively used the risk subjective perception data of road users in different interactive scenarios to mine potential safety risk factors to take measures to prevent accidents.

The remainder of this paper is structured as follows: Section 2 provides a brief overview of the literature related to mixed traffic safety; Section 3 describes the data collection; Section 4 details the data analysis model and results; Section 5 presents a discussion; and Section 6 presents conclusions and suggestions for future research.

## 2 Literature review

In recent years, the rapid development of autonomous driving technology has attracted widespread attention with respect to the traffic safety of AVs. Although AVs are in the testing phase and traffic accident samples are limited, several studies have been conducted to estimate crash rates involving AVs and analyze the factors contributing to the severity of accidents involving AVs [4]. The first paper that examined this topic used accident data on AVs from September 2014 to November 2015 in California and reported that the number of accidents was highly correlated with the number of autonomous miles traveled [12]. Favarò et al. also analyzed traffic accidents with AVs in California and obtained similar results, but only in different time ranges [13]. Tu et al. established a hierarchical Bayesian network structure to compare the influence of causes of AV road testing accidents with that of human-driving accidents. The results revealed that there were significant differences between the two types of accidents, and AV road testing had poor adaptability in complex traffic environments, poor horizontal and vertical road alignments, and low-light conditions [14]. Abdel-Aty and Ding also investigated the differential characteristics of autonomous versus human-driven vehicle accidents and suggested that accidents involving AVs occurred more frequently than HV accidents under dawn/dusk or turning conditions [3]. Xu et al. attempted to investigate property damage only (PDO) and non-PDO crashes on the basis of reports of AV crashes in California and reported that the AV driving mode, collision location, roadside parking, rear‒end collisions, and one-way streets were the major factors influencing the type and severity of AV collisions; however, this study did not consider class imbalance of data, which is highly important [15]. Wang and Li developed a CART model to estimate the risk factors for crashes involving AVs using AV accident reports in California from 2014-2018. The results indicated that highways were locations where severe injuries were likely to occur [16]. Das et al. identified six classes of collision patterns via a Bayesian latent class model and reported that a greater proportion of the injury severity level was associated with turning, multivehicle collisions, sideswipe and rear-end collisions, and dark lighting conditions with streetlights [17]. In addition, weather was confirmed as a factor affecting the severity of the AV‒involved accidents. Chen et al. used an XG-Boost model to identify key features that affect crash severity, including weather, vehicle damage, accident location, and crash type [8]. Liu et al. discovered that weather conditions, road design and traffic flow characteristics significantly impact real-time collision risk using a mixed logit model [18]. Another study revealed that clear weather conditions could reduce the likelihood of injurious collisions involving AVs [19].

In addition to the use of AV accident data, some studies have been conducted using traffic simulations. To assess real-time collision risk in a mixed traffic environment, Lu et al. proposed the kernel logistic regression (KLR) model to evaluate the crash risk in real-time [20]. Guériau and Dusparic conducted a comprehensive study using SUMO to assess the impact of CAVs on the efficiency and safety of three types of networks (urban, national, motorway), and the results revealed that conflicts improved with increasing CAV penetration rates [21]. The same conclusion was reached in another study. Arvin et al. employed SUMO to evaluate the safety of CAVs in mixed traffic at intersections. The results indicated that increasing the market penetration rate of CAVs reduced the number of conflicts, and optimal road safety could be achieved when the market penetration was 40%. In addition, VISSIM software was used to simulate the traffic operation of CAVs [22]. Papadoulis et al. developed a decision-making algorithm in VISSIM software and used a surrogate safety assessment model (SSAM) to analyze safety. The study revealed that traffic conflicts decreased as the penetration rate of CAVs increased, which was consistent with the conclusions of other studies [23].

Previous studies have generally focused on various contributing factors affecting the collision types and severity levels of AV-involved crashes based on real accident data and simulation data. The subjective risk perceptions of various traffic participants in mixed traffic environments and the potential risk impact of traffic operation have been largely overlooked. Moreover, the accident data used for the analysis were all from accident reports in California, ignoring discrepancies between different countries. To address the above limitations, this study investigates safety risks in mixed traffic environments from multiple dimensions. More specifically, this study employed the AV crash report in California due to the open database and available and enlarged variables related to roadway features by Google Street View. Moreover, this study investigated abnormal driving behavior data of AVs instead of accident data in China to explore the differences in accident risk between China and the United States. In addition to objective data, this study also analyzes the subjective risk perceptions of road users when they interact with AVs. The results of this study have the potential to provide comprehensive insight for improving mixed road traffic safety and promoting the development of AV industries.

## 3 Data preparation

### 3.1 Crash reports involving AVs

With the implementation of California Senate Bill 1298, the Department of Motor Vehicles (DMV) required that crash reports involving AVs be submitted within 10 business days of the crash occurrence [15]. The database is open to the public and available; hence, this study used a total of 290 AV-involved crashes between January 2019 and August 2022.

The information extracted from the crash reports includes crash severity, type of collision, driving mode of the autonomous vehicles, vehicle movement preceding collision, crash time, crash site, lighting, roadway conditions, roadway surface and weather. In addition, the detailed road infrastructure data for each crash were collected from Google Street View, including the type of road, slope, traffic control type, number of lanes and dividing medians.

The accidents are divided into two categories according to their severity: no damage accidents (31) and damage accidents (259). Seventeen variables were collected as independent variables from the vehicle motion state, environment and road features. Their descriptions are detailed in Table 1.

**Table 1:** Variable descriptions.

Variable category	Variable	Description
Crash severity	Vehicle damage	No damage = 0, Damage = 1
Vehicle movement	Disengagement	No = 0, Yes = 1
	Type of collision	Rear-end = 1, Broadside = 2, Sideswipe = 3, Head-on = 4
	AV movement preceding the collision	Straight = 1, Making left turn/Making right turn = 2, Making turn/ Passing other vehicle/ Changing lanes = 3, Stopped = 4
	Other vehicle movement preceding the collision	Straight = 1, Making left turn/Making right turn = 2, Making turn/ Passing other vehicle/ Changing lanes = 3, Stopped = 4
Environment	Workday	No = 0, Yes = 1
	Crash time	Off-peak = 0, Peak = 1
	Weather	Clear = 1, Adverse weather = 2
	Lighting conditions	Daylight = 0, Night = 1
	Roadway surface	Dry = 0, Wet = 1
Roadway	Roadway Condition	Normal = 0, Construction/Jam = 1
	Crash Location	Segment = 0, Intersection = 1
	Type of Traffic Control	No control = 1, Signal control = 2, Traffic sign = 3
	Slope	Low = 0, High = 1
	Curvature	Small = 0, Large = 1
	Type of road	One-way road = 0, Two-way road = 1
	Number of lanes	1-2 lanes = 1, 3-4 lanes = 2, 4 More than 4 lanes = 3
	Dividing medians	Undivided road = 1, Line separator = 2, Central median = 3

### 3.2 Survey of the abnormal driving behaviors of AVs

Since there is currently no open AV accident database in China, this study uses traffic conflicts to replace accidents for safety risk analysis. The research team investigated the abnormal driving behavior of AVs in the face of traffic conflicts. The observers rode driverless taxis and recorded abnormal behaviors such as braking and automatic vehicle disengagement through the “2bulu” app. The “2bulu” app is a mobile application that records outdoor travel trajectories. The survey area is the Wuhan Economic and Technological Development Zone, where the commercialization of driverless taxis was implemented. The survey lasted for 2 weeks, including off-peak hours, peak hours, weekdays, weekends, and both day and night. The data collected by observers include the type, location, time and cause of abnormal behavior. Additionally, the research team collected roadway information for each abnormal behavior from Baidu Maps, including the lane number and traffic control type, divided the data into medians and recorded the reasons for the occurrence of abnormal behavior. In total, 452 data points were collected, including 367 for emergency braking and 85 for disengagement.

**Fig 1:**
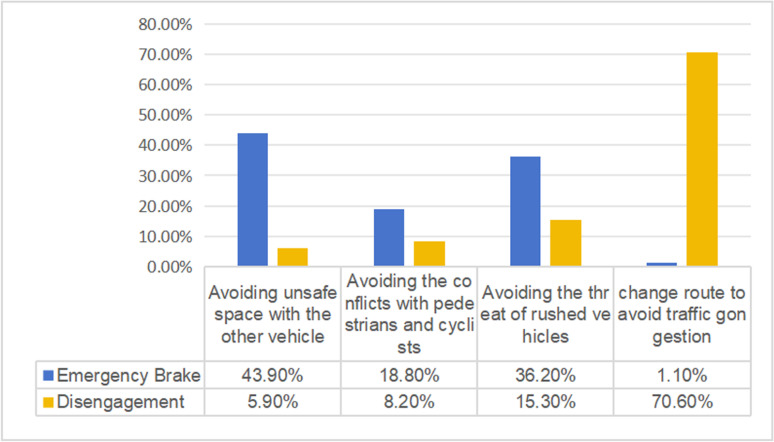
Reasons of abnormal driving behavior.

The survey revealed that the abnormal driving behavior of automatic vehicles was caused mainly by factors such as insufficient space with the vehicle ahead or the vehicles in adjacent lanes, conflicts with pedestrians and cyclists, the threat of rushed vehicles and traffic congestion (Fig. 1).

As disengagement was caused mainly by traffic congestion, this study discussed only the factors contributing to emergency braking. The data are divided into three categories according to the reasons for braking: avoiding unsafe space with other vehicles, avoiding conflicts with pedestrians and cyclists, and avoiding the threat of rushed vehicles. Ten variables were collected as independent variables, as shown in Table 2.

**Table 2:** Variable descriptions.

Variable category	Variable	Description
Braking reasons	Type of brake	Avoiding unsafe space with other vehicles = 1, Avoiding conflicts with pedestrians and cyclists = 2, Avoiding the threat of rushed vehicles = 3
Environment	Weather	Clear = 0, Cloudy and raining = 1
	Lighting	Daylight = 1, Dusk-dawn = 2, Dark-street lights = 3
	Time	Off-peak = 0, Peak = 1
	Weekday	No = 0, Yes = 1
	The other part involved	HV = 1, Pedestrian or Cyclist = 2, Emergency = 3
Roadway	Road classification	Expressway = 1, Arterial = 2, Collector = 3, Local street = 4
	Location	Crosswalk or access = 1, Intersection = 2, Approach of intersection = 3, Road segment = 4
	Number of lanes	Less than 4 lanes = 1, 5-6 lanes = 2, More than 6 lanes = 3
	Dividing medians	Undivided road = 1, Line separator = 2, Central median = 3
	Control Type	No control = 1, Signal control = 2, Traffic sign = 3

### 3.3 Questionnaire on the traffic behavior of traffic participants

To examine the subjective risk perceptions of road users when they interact with AVs, a survey questionnaire was designed to gather information from traffic participants regarding their attitudes toward automatic vehicles and their traffic behaviors in different interaction scenarios. The questionnaire was distributed in September 2023, and the submission of the completed questionnaire was considered to have provided informed consent. The survey for driverless taxi test operators focused exclusively on scenarios of active takeover and periods of driving fatigue. For drivers, pedestrians and cyclists, the questionnaire was designed in two parts based on the Driver Behavior Questionnaire (DBQ) [24]. The first part covered personal information, including their usual travel mode, age, gender, education, driving age, trust in automatic vehicles, and involvement in traffic accidents over the past three years. The second part addressed behavioral choices in some typical conflict scenarios involving automatic vehicles. Table 3 presents one of the questions about the conflict scenarios for pedestrians and cyclists asked in the survey.

**Table 3:** One of the conflict scenarios for pedestrians and cyclists asked in the survey.

Scenario 1: “You are about to cross an unsignalized crosswalk when a vehicle is quickly approaching the crosswalk.” How likely are you to rush across the street?
(a) If the vehicle is a human-driven vehicle: 1 (unlikely) 2 (slightly likely) 3 (moderately likely) 4 (likely) 5 (very likely)
(b) If the vehicle is an automatic vehicle: 1(unlikely) 2 (slightly likely) 3 (moderately likely) 4 (likely) 5 (very likely)

The questionnaire was distributed online to individuals in Wuhan, Hubei Province, China. Moreover, the research team also went to shopping malls and asked some residents of the Wuhan Economic and Technological Development Zone to complete the surveys. In total, 449 effective responses were collected, including 223 for pedestrians and cyclists, 194 for human drivers and 32 for driverless taxi test operators.

Owing to the information confidentiality requirements of driverless taxi test operators, this study reports only the personal information of other responders, as shown in Table 4. Most responders showed pessimistic attitudes toward autonomous vehicles. A total of 90.17% of the responders expressed concerns about AV technologies, and 80.34% of the responders trusted human drivers more in emergencies, which may be related to people’s lack of awareness or knowledge of AVs. We suggest that more information should be provided to the public to let them know more about the performance of AVs, which is conducive to the promotion of AVs.

**Table 4:** Descriptive statistics of personal information.

Content	Possible options	Distribution
Travel Mode	Walking, bicycle, and public transport	53.48%(223)
	Self-driving	46.52%(194)
Age	18-25	44.60%(186)
	25-40	35.97%(150)
	40-55	17.75%(74)
	>55	1.68%(7)
Gender	Male	61.63%(257)
	Female	38.37%(160)
Education	Middle school	2.16%(9)
	High school	10.79%(45)
	Bachelor degree or above	87.05%(363)
Concerns about automatic Vehicles	Not concerned	9.83%(41)
	Slightly concerned	69.06%(288)
	Moderately concerned	16.31%(68)
	Very concerned	4.80%(20)
Who do you think handle automatic vehicle in emergencies to make you feel safer?	Human driver	80.34%(335)
	AV system	19.66%(82)

## 4 Safety risk analysis of mixed traffic

### 4.1 Analysis of crash reports

#### 4.1.1 Balanced sample set.

Because the number of no-damage accidents in the current sample set is significantly less than the number of damage accidents, this may lead to the weak recognition ability of the model for no-damage accidents. The synthetic minority-over-sampling technique (SMOTE) is a data enhancement algorithm for addressing the class imbalance problem. It is an oversampling method that generates new samples by interpolation, thereby increasing the number of samples for the minority class. The method has the advantages of reducing the risk of overfitting and improving the performance of the model [25]. Hence, the SMOTE algorithm was employed in this study to address imbalanced datasets before modeling.

#### 4.1.2 Crash severity prediction model.

(1) XGBoost algorithm

The XGBoost model has been shown to be more accurate than other machine learning models (logistic regression, SVM, deep neural network, etc.) in predicting the likelihood of an accident [26]. The core of XGBoost is an integrated algorithm based on gradient-boosted decision trees. It utilizes a series of decision trees, where every tree learns from the prior tree and influences the following tree to promote model performance [8]. One of the advantages is the regularization of the loss function, which can effectively reduce the number of calculations of the model and prevent the model from overfitting, thus improving the efficiency of model training [27]. The objective function Obj for the kth iteration can be expressed by Equation (1):


$$Obj^{\left(t\right)}=\sum_{i=1}^{n}l\left(y_{i},{\hat{y}}_{i}^{\left(t\right)}\right)+\sum_{j=1}^{t}\Omega \left(f_{j}\right)$$ (1)

where *n* is the number of samples, ${{\hat{y}}_{i}}^{\left(t\right)}$ is the prediction value of the sample i at iteration t, and l is the original loss function. represents the regularization term, as shown in Equation (2).


$$\Omega (f)=\gamma T+\frac{1}{2}\lambda \sum_{j=1}^{T}\omega_{J}^{2}$$ (2)

Where, T is the number of leaf nodes, ω is the value of the leaf nodes, and γ and λ are two constants used to regulate the degree of regularization.

This study used a grid search and cross-validation to determine the best combination of parameters to prevent the model from overfitting. The final parameter values for the XGBoost model are presented in Table 5.

**Table 5:** Final parameter settings for the XGBoost model.

Parameter	Parameter meaning	Value
objective	The objective function	binary:logistic
n_estimators	The number of weak learners	1000
max_depth	The depth of the tree, controlling how deep each tree grows	9
reg_lambda	The L2 regularization term for weights	3
max_delta_step	The maximum step size for changing weights of each tree	2
min_child_weight	The minimum sum of sample weights needed in a child node	0.8
colsample_bynode	The proportion of features randomly chosen for each node during training	0.9
subsample	The proportion of samples used for training	0.3
learning_rate	The step size for updating weights in each iteration	0.01

Seventy percent of the data were randomly selected as the training set, whereas the remaining 30% were used for the test set. The divided training set and test set were subsequently input into the XGBoost model, random forest model and decision tree model. The performance estimation results of the models with balanced datasets and imbalanced datasets are listed in Tables 6 and 7.

**Table 6:** The performance estimation results of models with imbalanced data.

Data set	Model	Accuracy	Precision	Recall	Area under curve	F1-score
Training set	XGBoost	0.8851	0.8915	0.9914	0.513	0.9388
	Decision tree	0.6051	0.9638	0.5733	0.7004	0.7189
	Random forest	0.5842	0.9362	0.569	0.6293	0.7078
Test set	XGBoost	0.8621	0.9259	0.9259	0.463	0.9259
	Decision tree	0.3793	0.8462	0.4074	0.2037	0.5499
	Random forest	0.4483	0.8667	0.4815	0.2407	0.619

**Table 7:** The performance estimation results of models with balanced data.

Data set	Model	Accuracy	Precision	Recall	Area under curve	F1-score
Training set	XGBoost	0.925	0.937	0.911	0.925	0.924
	Decision tree	0.630	0.778	0.352	0.627	0.485
	Random forest	0.658	0.660	0.637	0.657	0.648
Test set	XGBoost	0.846	0.900	0.788	0.848	0.840
	Decision tree	0.590	0.711	0.338	0.596	0.458
	Random forest	0.654	0.697	0.575	0.656	0.630

The performances of the XGBoost model, decision tree model, and random forest model were compared in terms of accuracy, precision, recall, and F1 score. Higher values of accuracy, precision, and recall indicate better practical performance of the model. Additionally, the closer the F1 score is to 1.0, the better the prediction performance. The model’s predictive ability was further assessed using the area under the curve (AUC) of the receiver operating characteristic (ROC) curve. A higher AUC signifies stronger predictive power. A comparison of the five metrics before and after addressing class imbalance found that the XGBoost model outperformed the other two models across all the evaluation criteria (Tables 6 and 7).

To further investigate the impact of data imbalance, the performance of the XGBoost model was specifically compared before and after balancing the dataset. Comparisons were conducted on both the training set and the test set to comprehensively evaluate the model’s performance. The results revealed that, with the imbalanced dataset, the recall rate was high, but the AUC value was lower, suggesting that the model may have been biased toward the majority class. However, in the balanced dataset, the AUC value improved significantly, indicating enhanced classification performance and better generalization capability.

Based on these considerations, this study adopted the XGBoost model to analyze accident severity in depth with a balanced dataset.

(2) Shapley additive explanations (SHAP)

The problem of interpretability remains an issue for ensemble learning methods. The game theory-SHAP method addresses this issue effectively. The core principle of SHAP is to apply the Shapley value of cooperative game theory to the interpretation of machine learning models, and to evaluate the impact of each feature variable on the prediction results by calculating its marginal contribution relative to the baseline. The Shapley value is calculated using the following equation [28]:


$$\phi_{i}=\sum_{x\subseteq M\backslash \left\{i\right\}}\frac{\left|X\right|!\left(\left|M\right|-\left|X\right|-1\right)!}{\left|M\right|!}\left[f\left(X\cup \left\{i\right\}\right)-f\left(X\right)\right]$$ (3)

Where $\phi_{i}$ denotes the contribution of variable i, M represents the feature variable, X represents the feature combinations that exclude the feature i, and |M| and |X| denote the dimensionality of the respective features. f(X∪{i})−f(X) represents the marginal contribution.

#### 4.1.3 Results analysis.

(1) Analysis of the importance of accident factors

**Fig 2:**
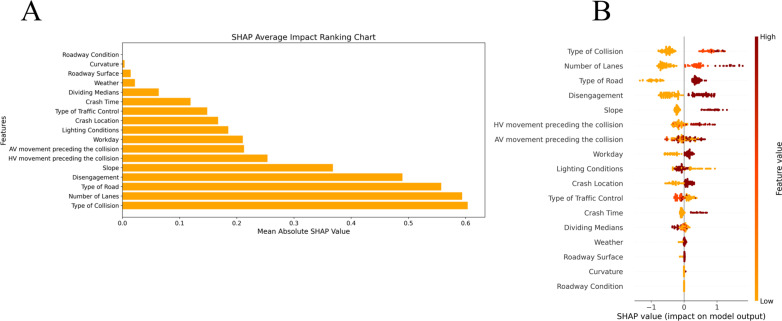
SHAP values for features of automatic vehicle accident. A) Feature variable importance plot. B) SHAP global analysis visualization.

The average SHAP values of the characteristic variables of the crash-involved AV were sorted to determine each characteristic variable’s contribution to the accident prediction result (Fig. 2). The collision type, type of road, and disengagement were found to be significant factors affecting the severity of accidents, which is consistent with previous research [8]. The difference is that this study finds the number of lanes to be the critical variable.

The visualization plot of SHAP (Fig. 2B) reflects the influence degree and direction of each characteristic variable on accident severity. The farther the point is from the centerline (zero), the greater the effect of that feature on the accident severity. A positive SHAP value indicates a positive effect, and a negative SHAP value indicates a negative effect. The redder the color is, the larger the value of the characteristic variable is. The more orange the color is, the smaller the value is. The type of collision has the greatest influence, whereas the road condition has the least influence (Fig. 2).

(2) Analysis of a single feature variable

**Fig 3:**
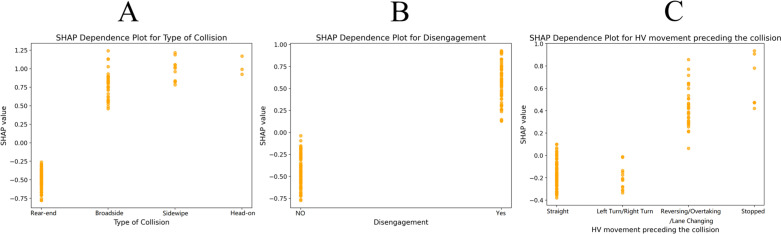
Analysis of single features of vehicle movement. A) Type of collision. B) Disengagement. C) HV movement preceding the collision.

**Fig 4:**
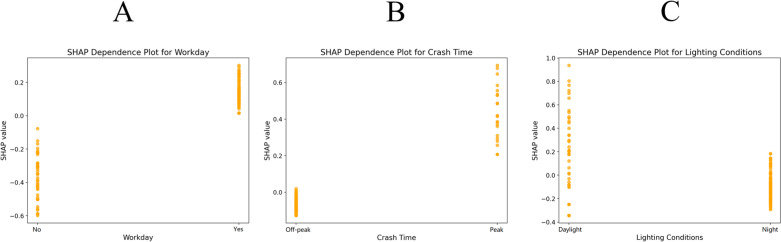
Analysis of single features under environmental attributes. A) Whether it is a workday. B) Crash time. C) Lighting conditions.

**Fig 5:**
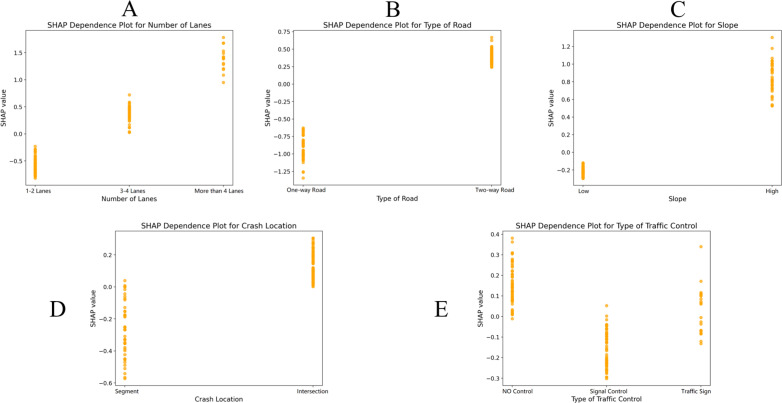
Analysis of single features under road attributes. A) Number of lanes. B) Type of road. C) Slope. D) Crash location. E) Type of traffic control.

To quantify the impact of individual feature variables on the severity of accidents, dependence plots were created to display the marginal effects of several variables. The SHAP dependency graphs illustrate feature variables related to vehicle movement, environment, and roadway (Fig 3–5).

The SHAP values of broadside, sideswipe, and head-on collisions were greater than 0, indicating that these three types of collisions increased accident severity (Fig 3). Similarly, AV disengagement and human vehicle movement preceding a collision, such as overtaking, changing lanes and slowing down, were associated with more serious accidents. Fig 4 shows that weekdays, peak hours and daylight increased the severity of accidents compared with weekends, off-peak hours, and nights. This may be related to the heavy traffic volume in those periods, which is consistent with the conclusions of existing research on traffic accidents involving autonomous vehicles [12]. When the traffic flow is high, the interactions between AVs and HVs are more frequent, which is more likely to lead to serious accidents. Fig 5 indicates that an increase in the number of lanes resulted in more serious accidents, possibly because the speed limits were higher on roads with more lanes, and a faster speed of collision would lead to more serious consequences. In addition, the number of crashes on two-way roads was greater than that on one-way roads, which is attributable to the collision types on two-way roads being generally head-on or sideswipe. Similarly, the consequences of accidents at intersections are more serious than those at road sections, which is also attributable to the collision types of accidents at intersections. In addition, high gradients and a lack of control could also worsen accidents.

### 4.2 Analysis of safety risk based on the abnormal driving behaviors of AVs

#### 4.2.1 Association rules.

The emergency brake behaviors of AVs can be attributed to many risk factors. To understand the relationships between factors, the association rule method was chosen for data mining and analysis. The Apriori algorithm, a classic association rule mining algorithm, offers simple operation and strong expansibility [29]. This study uses the Apriori algorithm to mine association rules for emergency brake data. The relevant formulas and concepts are defined as follows.

A rule can be defined as an implication, *X → Y*, where *X* is the event that occurs in the preceding item and *Y* is the event that occurs in the following consequent. This means that if *X* occurs in an emergency brake, *Y* will also occur.

Generally, there are three key indices in the Apriori algorithm, namely, support, confidence and lift. Support refers to the proportion of abnormal behaviors involving both *X* and *Y* in all emergency brakes, denoted by Sup(X→Y):


$$Sup\left(X\to Y\right)=\frac{count\left(X\cup Y\right)}{countD}$$ (4)

Confidence is the probability of *Y* occurring after the occurrence of event *X*, denoted Con(X→Y):


$$Con\left(X\to Y\right)=P\left(Y/X\;\right)\frac{count\left(X\cap Y\right)}{countX}$$ (5)

Lift indicates the elevating effect that event *X* has on the probability of the occurrence of event *Y*, denoted Lift(X→Y):


$$Lift\left(X\to Y\right)=\frac{con\left(X\to Y\right)}{SupY}$$ (6)

#### 4.2.2 Results Analysis.

In this study, an association rule analysis of emergency brake types was conducted to explore safety risk factors. On basis of previous studies that applied association rules to analyze traffic safety and enhance the strength and accuracy of association rules [30], the thresholds of the three indicators were carefully determined. With support ≥  10%, confidence ≥  70%, and lift ≥  1, 53 association rules for avoiding unsafe space with other vehicles were extracted. With support ≥  5%, confidence ≥  70%, and lift ≥  1, 57 association rules for avoiding conflicts with pedestrians and cyclists were identified. With support ≥  5%, confidence ≥  65%, and lift ≥  1, 55 association rules for avoiding the threat of rushed vehicles were extracted. The partial results are presented in Table 8.

**Table 8:** Strong association rules for different emergency brake types.

Post-condition	Strong association rule	Support	Confidence	Lift
Avoiding unsafe space with other vehicles	Night + No control + Segment + Road with central median	0.133	0.75	2.042
	Night + Peak hour + Road with central median + Workday	0.106	0.750	2.042
	Night + No control + Segment + Road with central median + Clear	0.133	0.750	2.042
	Night + No control + Road with central median	0.131	0.746	2.031
	Night + No control + Segment + Road with central median + Workday	0.131	0.746	2.031
	......	......	......	......
avoiding conflicts with pedestrians and cyclists	Arterial + Daytime	0.053	1.000	5.947
	Arterial + Workday	0.060	1.000	5.947
	Arterial + Clear	0.069	1.000	5.947
	Arterial + Workday + Clear	0.060	1.000	5.947
	arterial	0.093	0.976	5.806
	......	......	......	......
Avoiding the threat of rushed vehicles	Signal control + 5-6 lanes + Road with central median + Clear	0.064	0.724	2.242
	Signal control + Peak hour	0.055	0.720	2.229
	Signal control + Peak hour + Clear	0.055	0.720	2.229
	Signal control + 5-6 lanes + Segment + Workday	0.055	0.720	2.229
	Signal control + 5-6 lanes + Segment + Clear	0.055	0.720	2.229
	......	......	......	......

The following patterns can be derived from these strong association rules. (1) Emergency braking that avoids unsafe space with other vehicles is likely to occur at road segments with central medians at night, if there is no traffic control. (2) Emergency braking that avoids conflicts with pedestrians and cyclists often takes place on arterial roads in the daytime. (3) Emergency braking that avoids the threat of rushed vehicles is likely to occur on sunny days with signal control, medians and multilane sections.

**Fig 6:**
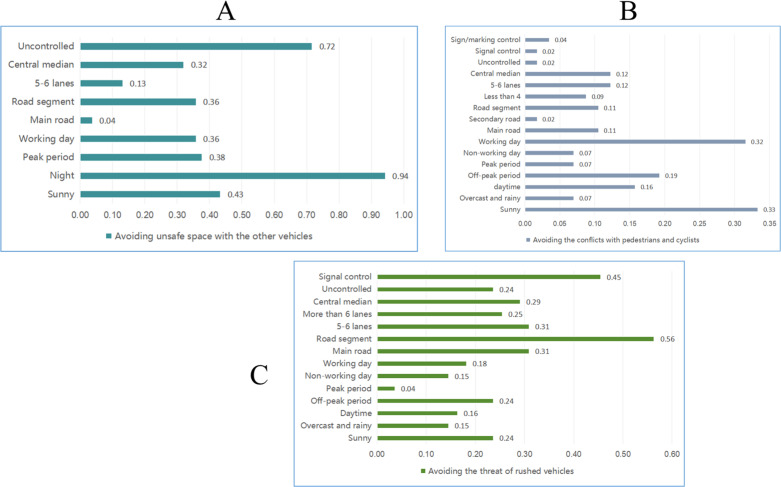
Frequency of associated causes for emergency brakes. A)Avoiding unsafe space with the other vehicles. B) Avoiding the conflicts with pedestrians and cyclists. C) Avoiding the threat of rushed vehicles.

Owing to the large number of strong association rules, the associated causes became scattered and difficult to enumerate, and it was found that the internal causes of different types of strong association rules varied; therefore, high-frequency factors were identified by calculating the frequency of various causes in strong association rules. The frequency of associated causes of different brake types is illustrated in Fig 6.

Clear days, weekdays, road sections, 5-6 lanes, and roads with central medians are the common strong association factors for the three types of sudden braking, which indicates that emergency braking is prone to occur under these road conditions, and that the driving risk is high (Fig. 6). This may be related to the heavy traffic flow and the frequent interaction between vehicles on weekdays, as well as the higher likelihood of lane changes on multilane roads. Additionally, “ghost pedestrian or vehicle” incidents were more likely to occur on roads with central medians, possibly because autonomous vehicles cannot detect ghost people or vehicles because of obstruction by the central divider, thus resulting in sudden braking. Hence, measures to improve the information detection ability of AVs, such as advanced sensors and the V2X technique, are necessary. For emergency braking, avoiding unsafe space with other vehicles, night and uncontrolled road sections are significant causes. This may be attributed to the faster speed on the uncontrolled road and the weaker lighting conditions at night, which may cause the vehicle to initiate hard braking when it encounters a sudden situation such that the following autonomous vehicle also brakes hard. For emergency braking to avoid conflicts with pedestrians and cyclists, the most frequent strong association factors are “clear” and “weekday.” This is mainly because on clear weekdays, the number of pedestrians and cyclists may increase, and the interaction between people and automatically driven vehicles is frequent, which can lead to sudden braking of autonomous vehicles. For emergency braking to avoid the threat of rushed vehicles, road sections and signal control are notably frequent causes. This may be related to aggressive behavior towards autonomous vehicles by human-driven vehicles on multilane roads.

### 4.3 Analysis of the traffic behavior of traffic participants

#### 4.3.1 Traffic behavior of driverless taxi test operators.

**Fig 7:**
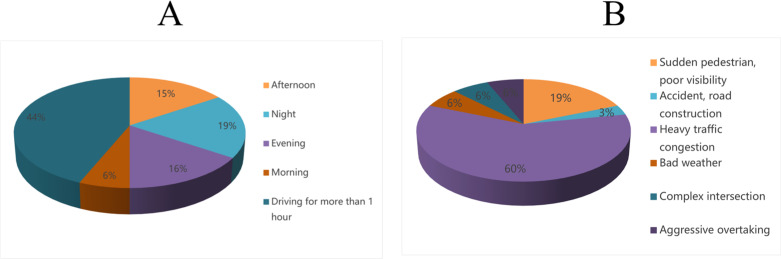
Survey statistics of safety operator. A) Responses to fatigue periods. B) Responses to takeover scenarios.

A statistical analysis of the responses to the driving fatigue and takeover scenarios was conducted (Fig 7). It was found that 43.8% of operators felt bored and tired after driving for an hour, indicating that extended periods of inactivity and the need for constant attention to road conditions can lead to fatigue in the morning compared with other times (6.3%), which may be due to individuals being more energetic and in better overall physical condition in the morning.

The main factors leading operators to take over the vehicle are heavy traffic and traffic congestion (59.4%), which aligns with the findings from the survey of abnormal driving behaviors. Sudden situations such as “ghost vehicles” and poor visibility due to vegetation accounted for 18.3%, indicating that in unexpected situations, autonomous vehicles still necessitate intervention from safety operators to make appropriate risk decisions. Adverse weather, intersections, and malicious overtaking by other vehicles each accounted for 6.3%. Adverse weather conditions may affect the perception of automatically driven vehicles and introduce driving risks. Frequent interactions between automatic vehicles, manual vehicles and pedestrians at intersections, as well as malicious behavior by human-driven vehicles, pose certain risks to automatic vehicles.

#### 4.3.2 Traffic behavior of pedestrians and drivers.

(1) Average aggressiveness degree

To quantify the risk of various traffic scenarios under mixed traffic flow and explore pedestrians’ and drivers’ aggressive behavior choices in different scenarios, this study proposed the concept of the average degree of aggressiveness. This metric quantifies the likelihood of participants engaging in aggressive behaviors such as speeding, cutting in, and overtaking in various traffic conflict scenarios.

The probability of aggressive behavior is assessed using a scale ranging from 1 to 5 (i.e., 1 =  unlikely; 2 =  slightly likely; 3 =  moderately likely; 4 =  likely; 5 =  very likely). The average aggressiveness degree is calculated as the weighted sum of the scores ranging from 3 to 5 for aggressive behaviors in various traffic scenarios divided by the total number of scores. This is expressed in Equation 7.


$$D=\frac{\sum {\mathrm{\backslash nolimits}}_{i=3}^{5}in_{i}}{\sum {\mathrm{\backslash nolimits}}_{i=3}^{5}n_{i}}$$ (7)

Where i represents the score given by each participant and where $n_{i}$ denotes the number of scores that equal i.

A lower average aggressiveness degree indicates a higher perceived safety risk in the scenario by traffic participants and reflects more conservative driving behavior.

(2) Statistical analysis

To determine whether the differences in risk perceptions between autonomous and human-driven vehicles across different respondents are statistically significant, statistical analyses were conducted on the dataset collected through a questionnaire survey. Specifically, paired-samples *t* tests were employed to compare the datasets of risk perceptions related to autonomous versus human-driven vehicles. The *t* value represents the test statistic, and after the *t* value is calculated, the corresponding *p* value can be obtained by referencing a *t* distribution table. For the same degrees of freedom, the larger the *t* value is, the smaller the *p* value. A small *p* value (typically less than 0.05) suggests that the observed difference is statistically significant. However, a small *p* value may reflect only a large sample size, and effect size analysis can provide insight into the practical significance of the observed difference. The most common effect size measure, Cohen’s d, quantifies the magnitude of the difference or association between variables observed in the study. A Cohen’s d value of 0.2 ≤  *d* <  0.3 indicates a small difference, 0.3 ≤  *d* <  0.5 indicates a medium difference, and *d* ≥  0.5 indicates a large difference. Additionally, analysis of variance (ANOVA) was used to assess whether there were differences between the various scenarios. If such differences were found, Tukey HSD tests were conducted to further evaluate the differences in risk perceptions among respondents across different scenarios. These analyses were used to validate the rankings of aggressiveness across various scenarios, ensuring that the observed rankings are statistically supported.

(3) Traffic behavior analysis of pedestrians and cyclists

The survey of pedestrian traffic behavior was structured around three distinct traffic scenarios. The average aggressiveness degree of pedestrians, when encountering autonomous and human-driven vehicles in potentially dangerous traffic scenarios, was assessed and is presented in Table 9.

**Table 9:** Average aggressiveness of pedestrians and cyclists in various scenarios.

Traffic scenarios	Facing automatic vehicles	Facing manual vehicles
**Scenario 1:**“You are about to cross an unsignalized crosswalk when a vehicle is quickly approaching the crosswalk.” How likely are you to rush across the street?	3.54	3.61
**Scenario 2:**“During morning and evening peak hours, there is a queue of vehicles in front of crosswalk. You are about to cross, but the pedestrian green signal will withdraw.” How likely are you to cross the street quickly?	3.73	3.68
**Scenario 3:**“During off-peak hours, you are preparing to cross the crosswalk while the pedestrian red light is on and a vehicle is approaching.” How likely are you to run a red light and cross the street?	3.48	3.59

To explore differences in risk perception between pedestrians facing human-driven and autonomous vehicles, an independent samples *t* test was conducted. The results revealed that the difference in risk perception was not statistically significant (*t* =  0.83, *p* =  0.4524). However, effect size analysis indicated a medium effect (Cohen’s d =  0.44), suggesting a meaningful difference between the two groups despite the lack of statistical significance. This result may be attributed to the small sample size and limited statistical power. Future studies should address these limitations by increasing the sample size or improving the experimental design to validate this trend.

In terms of aggressiveness, pedestrians are more aggressive in scenario 2, likely because they are more inclined to cross the street when the light is green. Conversely, in Scenario 3, individuals are less inclined to cross the street when the light is red. The average degree of aggressiveness in Scenario 1 is lower than that in Scenario 2, likely because pedestrians perceive the unsignalized control scenario as more hazardous and consequently adopt more conservative behaviors. These observed differences in aggressiveness across the scenarios were statistically validated through Tukey HSD tests (Table 10). Specifically, the risk perception level in Scenario 2 was significantly lower than that in Scenario 1 (mean difference =  -0.4709, *p* =  0.0001) and Scenario 3 ((mean difference =  0.6547, *p* =  0.0001), indicating that pedestrians in Scenario 2 demonstrated higher levels of aggressiveness.

**Table 10:** Tukey HSD test for comparing aggressiveness of pedestrians and cyclists across scenarios.

Group1	Group2	Mean difference	p-adj
**Facing automatic vehicles**			
Scenario 1	Scenario 2	0.4709	0.0001
Scenario 1	Scenario 3	-0.1839	0.2457
Scenario 2	Scenario 3	-0.6547	0.0000
**Facing manual vehicles**			
Scenario 1	Scenario 2	0.6054	0.0000
Scenario 1	Scenario 3	-0.1121	0.6084
Scenario 2	Scenario 3	-0.7175	0.0000

Furthermore, pedestrians are more conservative in the face of automated vehicles than in the face of manual vehicles, except in Scenario 2, which is different from the findings of a previous study [31]. This finding indicates that pedestrians perceive interactions with autonomous vehicles as more dangerous than interactions with manual vehicles do. The result in Scenario 2 is an exception, possibly because individuals believe that autonomous vehicles adhere more strictly to traffic rules than manual vehicles do.

(4) Traffic behavior analysis of drivers

The survey of driver traffic behavior included nine scenarios. The average degree of aggressiveness of drivers in various scenarios is presented in Table 11.

**Table 11:** Average aggressiveness degree of drivers in various scenarios.

Traffic scenarios	Facing automatic vehicles	Facing manual vehicles
**Scenario 1:**“when the green signal is about to end, you are turning left, meanwhile an opposite vehicle is going straight through the intersection. “ If you judge that there is a chance to rush to turn left, how likely are you to rush to cross the opposite vehicle before the signal turns red?	3.49	3.58
**Scenario 2:**“in the green light signal, you follow the vehicles in front to turn left, and the vehicle in front stops in the intersection to avoid the opposite straight vehicle. “ If you judge that you have the chance to bypass the front car to rush through, how likely are you to rush through?	3.62	3.63
**Scenario 3:**“You follow the vehicles in front on a multi-lane road and encounter a bottleneck where the number of lanes decreases.” If you judge that you have the opportunity to change lanes, how likely are you to choose to change lanes?	3.46	3.65
**Scenario 4:** “At a crosswalk and road access ahead, the vehicle in front of you keeps yielding.” If you judge that you have the opportunity to change lanes, how likely are you to choose to change lanes?	3.57	3.7
**Scenario 5:**“you travel on the road and encounter slow-moving traffic ahead.” If you judge that you have the chance to change lanes, how likely are you to change lanes and overtake facing AV and HV on the adjacent lane respectively?	3.71	3.71
Assuming that there is a self-driving vehicle in the same lane ahead you, if you judge that you have the opportunity to change lanes and overtake, how likely are you to change lanes and overtake in the following scenarios?		
**Scenario 6:**“During peak traffic periods or congested road sections.”	3.61	/
**Scenario 7:**“During off-peak hours or low flow periods.”	3.96	/
**Scenario 8:**“At night with no lighting and obstructed vision due to vegetation.”	3.39	/
**Scenario 9:**“In adverse weather conditions.”	3.51	/

The independent samples t test revealed a significant difference in the risk perception scores of drivers facing HVs and AVs (*t* statistic =  3.3714, *p* value =  0.0434).

In terms of aggressiveness, the average degree of aggressiveness of drivers interacting with automatic vehicles is lower than that of drivers interacting with manual vehicles. This finding indicates that drivers perceive interactions with autonomous vehicles as more hazardous, which correlates with lower levels of trust in automatic vehicles. This finding is consistent with the results from the above survey analysis in Section 3.3.

When interacting with AVs, drivers’ average degree of aggressiveness in Scenario 3 is the lowest (3.46), likely because the perceived high risk in bottleneck areas where narrow road space increases the likelihood of rear-end and sideswipe collisions. In contrast, Scenario 5 has the highest degree of aggressiveness (3.71), which was statistically validated through Tukey HSD tests (Table 12). Specifically, the mean difference between Scenario 5 and all other scenarios is negative, with *p* values less than 0.05. This finding indicates that in Scenario 5, where drivers perceive weaker risk, they are more likely to engage in risky driving behaviors, such as overtaking traffic congestion or slow-moving traffic.

**Table 12:** Tukey HSD Test for Comparing Aggressiveness of Drivers Across Scenarios.

Group1	Group2	Mean difference	p-adj
Facing automatic vehicles			
Scenario 1	Scenario 2	0.0412	0.9974
	Scenario 3	0.0464	0.9958
	Scenario 4	0.2062	0.4584
	Scenario 5	0.6082	0.0000
Scenario 2	Scenario 3	0.0052	1.0000
	Scenario 4	0.1649	0.6729
	Scenario 5	0.5670	0.0001
Scenario 3	Scenario 4	0.1598	0.6988
	Scenario 5	0.5619	0.0001
Scenario 4	Scenario 5	0.4021	0.0108
Facing manual vehicles			
Scenario 1	Scenario 2	-0.1031	0.9245
	Scenario 3	0.0309	0.9992
	Scenario 4	0.1649	0.6839
	Scenario 5	0.5103	0.0005
Scenario 2	Scenario 3	0.1340	0.824
	Scenario 4	0.2680	0.2074
	Scenario 5	0.6134	0.0000
Scenario 3	Scenario 4	0.1340	0.824
	Scenario 5	0.4794	0.0014
Scenario 4	Scenario 5	0.3454	0.0481

Additionally, among the four complex road environment scenarios, the drivers’ average degree of aggressiveness increases from Scenario 8 (3.39) to Scenario 7 (3.96). In Table 13, the average differences between Scenario 7 and Scenarios 6, 8, and 9 are -1.0722, -1.4845, and -1.7062, respectively, with *p* values of 0.000, 0.000, and 0.000, respectively. This suggests that drivers in Scenario 7 have weaker risk perceptions and are more likely to exhibit aggressive behavior. This finding indicates that in mixed traffic flows, drivers perceive greater risks in scenarios characterized by poor lighting at night, obstructed vision due to vegetation, adverse weather, and high traffic density during peak periods.

**Table 13:** Tukey HSD Test for comparing driver aggressiveness in complex scenarios with autonomous vehicles.

Group1	Group2	Mean difference	p-adj
Scenario 6	Scenario 7	1.0722	0.0000
Scenario 6	Scenario 8	‒0.4124	0.0028
Scenario 6	Scenario 9	‒0.6340	0.0000
Scenario 7	Scenario 8	‒1.4845	0.0000
Scenario 7	Scenario 9	‒1.7062	0.0000
Scenario 8	Scenario 9	‒0.2216	0.2371

## 5 Discussion

This study utilized three types of data: objective accident, conflict and subjective feeling data. In addition, the data collection area covered the United States and China. The results of the multidimensional analysis indicated that the risk factors affecting mixed traffic safety are related primarily to traffic conditions, road conditions, and the environment, as shown in Table 14.

**Table 14:** Safety risk factors of mixed traffic.

Data resources		Key risk factors	Consistent factors	Contrary factors
Objective data	Crash reports involving AV	**Traffic attributes:** AV Disengagement, HV making around, overtaking preceding collision, changing lanes preceding collision **Road attributes:** multiple lanes, two-way roads, intersection, high gradients, lack of control **Environment and time attributes:** daylight, weekdays, peak-hours	weekday, road section, multiple lanes, road with central medians, lack of control	daylight
Objective data	Abnormal driving behaviors	**Road attributes:** Uncontrolled, road section, central median, multiple lanes and signal control **Environment and time attributes:** clear, at night, weekdays		At night
Subject feelings	Questionnaire on traffic participants	**Traffic attributes:** Heavy traffic flow **Road attributes:** unsignalized, bottleneck section **Environment and time attributes:** peak periods, adverse weather, at night, poor vision due to vegetation	/	/

In terms of traffic conditions, factors such as AV disengagement, sudden maneuvers by human-driven vehicles, overtaking preceding collisions, changing lanes before a collision, and heavy traffic flow all contribute to an increased risk of safety in mixed traffic. Therefore, it is necessary to educate drivers to regulate their driving behavior and strengthen traffic management measures, especially when the traffic flow is heavy. For road conditions, factors such as multiple lanes, road segments, intersections, central medians, and lack of control contribute to increased mixed traffic safety risks. Hence, the safety design and management of these facilities should be improved. With respect to the road environment, factors such as lighting, weekdays, peak hours, and weather conditions also contribute to increased mixed traffic safety risks. By analyzing California’s AV crash reports using the XGBoost model, key factors such as collision type, number of lanes, road directionality (single or bidirectional), and whether the vehicle was in overtaking mode were identified as crucial variables affecting the severity of accidents. However, owing to the lack of publicly available AV accident data in China, this study employed traffic conflict surveys as a feasible alternative for assessing accident risk. The common key risk factors identified from the analysis of both objective data sources include weekdays, road sections, multiple lanes, roads with central medians, and lack of control. Notably, inconsistencies exist between the results of the crash report analysis and those of the abnormal driving behavior investigation, which may be attributed to several factors, including dataset differences, a small sample size of crash reports during the COVID-19 pandemic period, and regional variations. In the analysis of AV crash reports from California, a greater likelihood of dangerous accidents was observed during daylight hours, whereas in the analysis of abnormal driving behavior in China, safety risks were more prominent at night. This discrepancy may be because a significant portion of autonomous driving accident data from the U.S. occurred during the day. It may also be influenced by differences in road environments, weather conditions, and other factors, which can affect the perception and decision-making capabilities of autonomous vehicles in different scenarios. Improving the safety of autonomous vehicles in complex environments requires a comprehensive approach that involves advanced sensors, robust algorithms, and smart design considerations. Additionally, the qualitative data obtained from the survey effectively supported and validated the objective analysis results. The analysis of participants’ perceptions confirmed that road segment, nighttime conditions, and lack of control are key factors contributing to mixed traffic safety risk. Hence, we suggest improving lighting conditions or enhancing weather and lighting sensors. Moreover, traffic management departments should take management measures to address these risk factors.

Different methods revealed varying details of traffic safety risk. The XGBoost model is capable of handling large datasets and providing accurate predictions; the Apriori algorithm uncovers more latent risk factors and the underlying associations between them. Compared with the first two methods, which focus primarily on objective data, the survey reflects the subjective perceptions of the respondents, thereby enhancing the credibility and comprehensiveness of the research. Together, these three analysis perspectives provide a multidimensional, comprehensive framework for analyzing the safety risk factors in mixed traffic with autonomous vehicles.

## 6 Conclusions

This study analyzed the traffic safety risks associated with the mixed driving of human-driven and autonomous vehicles from multiple perspectives. The key conclusions are as follows.

(1) Risk factors contributing to crashes involving autonomous vehicles were identified using the XGBoost algorithm. The results indicated that types of collisions, such as “sideswipe”, “broadside” and “ head-on”, as well as environmental attributes, including “weekdays”, “peak periods” and “daylight”, and road attributes, such as “multiple lanes”, “two-way roads”, “high gradients”, “intersections” and “lack of traffic control”, were associated with a greater likelihood of injury accidents.
(2) The Apriori algorithm was used to identify risk factors associated with the abnormal driving behaviors of automatic vehicles in Wuhan, China. The results indicated that factors such as the “presence of manual vehicles”, “road segments”, “clear days”, “weekdays”, “central medians”, “5-6 lane roads” and “main roads” were more likely to lead to emergency braking.
(3) A survey of traffic participants was conducted to assess traffic safety behaviors. The results revealed that pedestrians exhibited more conservative behavior at uncontrolled crosswalks and during peak periods. Under mixed traffic conditions, drivers perceived higher risks at night, with poor lighting, reduced visibility due to vegetation, adverse weather, and peak periods.
(4) Based on the analysis results, we recommend some suggestions for safety improvement. It is important to educate the population about the characteristics of AVs, which can increase the population’s awareness of AV dynamic characteristics in traffic flows. Moreover, it is necessary to strengthen the education of drivers and pedestrians and regulate their traffic behavior. In addition, enhancing the environmental sensory ability and response sensitivity of automatic vehicles are the keys to improving the safety of mixed traffic. Improving road infrastructure design and traffic management can also promote the safety of mixed traffic flows.

As automated vehicles are in the early stages of development, the number of such vehicles on the road is relatively small, coupled with the COVID-19 pandemic, resulting in a limited sample size of AV accidents, which may introduce some deviations in the research results. Consequently, we recommend extending this study with new data continuously collected and appended to the database. Additionally, as abnormal driving behaviors are assessed manually, the accuracy of the data may need further improvement. Furthermore, respondents displayed relatively conservative attitudes during the survey, leading to discrepancies between the survey results and actual traffic operations. The sample size should be expanded to provide more generalizable and convincing results in future research.

In addition, despite the breakthroughs in traffic accident causation analysis achieved by the XGBoost model, several limitations remain. We observed a certain degree of performance variation between the training set and the test set, indicating the need for further research to ensure that the model performs effectively across a broader range of datasets. Moreover, future research could explore the synergy between different analytical methods and examine how integrating various datasets could improve the accuracy and practical significance of the analysis. By combining these approaches, it may be possible to develop more comprehensive and reliable models for AV-related safety and performance.

## Supporting information

S1 Data Raw Data. (XLSX)
