# Correction: Analysis of safety risks in mixed driving of manual and automatic vehicles: Multiple perspectives

**DOI:** 10.1371/journal.pone.0334074

**Published:** 2025-10-10

**Authors:** The PLOS One Staff

## Notice of Republication

This article was republished on September 22, 2025, to correct an error in the title. The word “Automatic” is misspelled in the article title. The correct title is: Analysis of safety risks in mixed driving of manual and automatic vehicles: multiple perspectives. The publisher apologizes for the error.

## Supporting information

S1 File Originally published, uncorrected article. (PDF)

S2 File Republished, corrected article. (PDF)
